# Data quality assessment and subsampling strategies to correct distributional bias in prevalence studies

**DOI:** 10.1186/s12874-021-01277-y

**Published:** 2021-04-30

**Authors:** A. D’Ambrosio, J. Garlasco, F. Quattrocolo, C. Vicentini, C. M. Zotti

**Affiliations:** Department of Public Health and Paediatric Sciences, University of Turin, Torino, Italy

**Keywords:** Healthcare associated infections, Prevalence studies, Sampling, Data quality, Methodology, Bias correction

## Abstract

**Background:**

Healthcare-associated infections (HAIs) represent a major Public Health issue. Hospital-based prevalence studies are a common tool of HAI surveillance, but data quality problems and non-representativeness can undermine their reliability.

**Methods:**

This study proposes three algorithms that, given a convenience sample and variables relevant for the outcome of the study, select a subsample with specific distributional characteristics, boosting either representativeness (Probability and Distance procedures) or risk factors’ balance (Uniformity procedure). A “Quality Score” (QS) was also developed to grade sampled units according to data completeness and reliability.

The methodologies were evaluated through bootstrapping on a convenience sample of 135 hospitals collected during the 2016 Italian Point Prevalence Survey (PPS) on HAIs.

**Results:**

The QS highlighted wide variations in data quality among hospitals (median QS 52.9 points, range 7.98–628, lower meaning better quality), with most problems ascribable to ward and hospital-related data reporting. Both Distance and Probability procedures produced subsamples with lower distributional bias (Log-likelihood score increased from 7.3 to 29 points). The Uniformity procedure increased the homogeneity of the sample characteristics (e.g., − 58.4% in geographical variability).

The procedures selected hospitals with higher data quality, especially the Probability procedure (lower QS in 100% of bootstrap simulations). The Distance procedure produced lower HAI prevalence estimates (6.98% compared to 7.44% in the convenience sample), more in line with the European median.

**Conclusions:**

The QS and the subsampling procedures proposed in this study could represent effective tools to improve the quality of prevalence studies, decreasing the biases that can arise due to non-probabilistic sample collection.

**Supplementary Information:**

The online version contains supplementary material available at 10.1186/s12874-021-01277-y.

## Background

Healthcare-associated infections (HAIs) and antimicrobial resistance (AMR) have been widely recognized as a significant threat to public health, with an estimated prevalence in acute care hospitals of 5.9% and an annual incidence of 3,760,000 new cases per year [1]. Therefore, a considerable amount of resources have been invested to monitor HAI prevalence and investigate the associated risk factors, with the objective of developing targeted intervention strategies. Several epidemiologic and surveillance studies have been conducted, ranging from hospital level [2], to country level [3, 4] to worldwide initiatives [5].

The European Union is on the frontline in monitoring and controlling HAI risk in its Member States, under the coordination of the European Centre for Disease Prevention and Control (ECDC) healthcare-associated infections surveillance network (HAI-Net). This network has carried out two European-wide Point Prevalence Surveys (PPSs), applying a standardized protocol developed by the ECDC, in 2011–2012 and 2016–2017 [6]. The aims of these studies were mainly to estimate HAI and antibiotic use prevalence in acute care hospitals, and to describe patients, infections, invasive procedures, antibacterial agent use and antimicrobial resistance, while also gathering information on hospitals’ characteristics and infection control practices.

In order to obtain robust estimates and to ensure comparability among participating countries, it is important to apply a consistent hospital sampling strategy. Therefore, the ECDC required every country participating in the PPS to provide a specific number of hospitals. This number is calculated to have a similar prevalence estimation error, taking into account the per-country hospital size distribution. Further, the ECDC required that the hospitals should be selected preferentially through systematic sampling among all hospitals in the country [6] taking into account hospital size. Countries were then categorized according to their ability to strictly follow the protocol in terms of sampling strategy and number of participating hospitals. Nevertheless, in the 2011–2012 PPS, sixteen out of thirty-three countries were not able to select hospitals through systematic sampling or provided data from fewer hospitals than requested by the ECDC [7]. When systematic sampling was not feasible, countries resorted to convenience sampling [8].

For the 2016 Italian PPS [9], convenience sampling was employed. The survey saw the participation of 135 hospitals altogether but, as indicated by the ECDC, a sample of 55 hospitals was required for the Italian sample. Generating a representative sub-sample from the 135 participating hospitals proved to be challenging for several reasons. First of all, regional participation in the survey was extremely heterogeneous, as the majority of hospitals were provided by two regions, Piedmont and Emilia-Romagna. Secondly, there was an excess of large hospitals in the sample compared to the actual size distribution of hospitals in Italy.

To address these issues, we developed three alternative, non-conventional sampling procedures for prevalence studies, that allow selecting a subset of units from a convenience sample. Furthermore, we developed a “Quality Score” which evaluates participating units according to data quality and completeness and can be used as an additional selection criterion.

The objective of these methodologies is to improve the representativeness and quality of epidemiologic studies when the selection of participating units is made using non-probabilistic strategies.

Data collected through the 2016 Italian PPS on HAIs was used to test and evaluate these methodologies, but their implementation is general and can be applied in a number of contexts.

## Methods

### Data sources

During the 2016 Italian PPS , 135 hospitals were selected by regional coordinators according to their availability to participate (convenience sample). Data were collected through a specific software developed by the ECDC (HelicsWin.Net) and installed locally in each participating hospital [10]. Reference personnel were trained prior to the study in HAI case definitions and data acquisition according to the ECDC PPS Protocol v5.3 [6]. Data were extracted locally from the ECDC software and sent to the Operational Contact Point at the Department of Public Health Sciences and Pediatrics, University of Turin, where they were merged and analyzed. Collected data included presence of HAI or antimicrobial usage (AMU) on the day of the survey, characteristics of patients, HAIs and AMU (if any). Furthermore, data on structural characteristics, organizational details and infection and AMR control measures and practices were collected at the ward and hospital level [6]. An open database available on the Italian Ministry of Health’s website, updated in 2018, was used as reference regarding the number of beds and the geographical distribution of Italian hospitals [11]. We made the version of the data used in this work available in the Github repository of the manuscript at: https://github.com/AD-Papers-Material/SubsamplingMethods/tree/master/Data.

### Quality score

A Quality score (QS) was developed with the objective of stratifying hospitals according to data completeness and quality.

The score was obtained by summing up a number of data quality indicators at the hospital level (full list available in Supplementary Material S1), chosen after evaluation of the collected data and identification of common errors and pitfalls. The indicators were further weighted for the “importance” of the involved variables, computed as the statistical association of each variable with the outcome of interest (i.e., HAI risk). Specifically, the weight was defined as ${10}^{\left(1-p. valu{e}_{LRT}\right)}$ with *p*. *value*_*LRT*_ being the *p*-value of a likelihood ratio test for a univariate logistic regression model predicting HAI risk given the variable, against an intercept-only model. The resulting score is a value between 1 and 10. For variables for which the statistical association with HAI risk was not computable (e.g., HAI characteristics, ECDC software warnings, patient admission dates), ad-hoc scores were assigned according to an evaluation of the importance of each variable with respect to the study.

The sum of these weighted indicators at the hospital level represents its final QS, with higher values indicating lower data quality.

An in-depth description of the methodology is described in Supplementary Material S1.

### Sampling procedures

Three sampling methodologies which generate subsamples with specific distributional characteristics were developed, starting from an initial collection of observational units collected through convenience sampling. The “*Distance procedure*” and the “*Probability procedure*” are aimed at producing subsamples that are more representative of the target population, while the “*Uniformity procedure*” tries to create subsamples that are uniform regarding specific characteristics of interest.

The procedures take into account a subset of characteristics which are relevant for the outcomes of interest. In this study, geographical location and hospital size were used, considering that these are known risk factors for HAI and increased AMU [1, 12], and that they are available at the country level. Concerning geographical location, hospitals were grouped by Italian region, while regarding hospital size, hospitals were grouped into 10 quantiles according to the number of beds for the Probability and Distance procedures and four quantiles for the Uniformity procedure. We used a higher number of quantiles for the first two procedures since it was more important to have a finer definition of the distribution of hospital sizes, while still retaining a meaningful number of hospitals in each size group. These groups are used to cluster hospitals into “size-location blocks” which are used to evaluate their distribution. Furthermore, all three procedures also consider the QS as an additional parameter for hospital selection. The Distance and Probability methods further require reference data regarding the target population; for this purpose, the Italian Ministry of Health’s database of all hospitals in the country, which reports hospital size and geographical position, was used [11]. For the purpose of the present study, the procedures were applied to the Italian 2016 PPS convenience sample of 135 hospitals to select a subsample of 55 units, as required by the ECDC protocol [6].

Here we provide a general description of the procedures while the detailed implementation and corresponding R code is presented in Supplementary Material S2. The code is also available online at https://github.com/AD-Papers-Material/SubsamplingMethods.

#### Uniformity procedure

This procedure acts iteratively selecting one hospital for each size-location block, taking the one with the lower (better) QS for each block, starting over until the required sample size is achieved. This produces a subsample with a similar number of hospitals for each block.

#### Probability procedure

Hospitals are allocated in order to obtain a final distribution of the size-location blocks in the subsample similar to the distribution present at the target population level: this distribution was derived after grouping all the hospitals in the country into the same blocks, according to the national hospital database. The allocation is achieved by sorting the hospitals according to the probability of the relative block at the national level weighted by the QS, and then selecting the required sample size among the units with the higher score.

#### Distance procedure

This procedure is useful when some of the distributional blocks are too underrepresented in the convenience sample; the procedure oversamples hospitals with characteristics similar to the underrepresented ones. In our algorithm, “Distance” is a numeric index defined as either the distance between Italian regions, divided into 5 groups according to a north-south gradient (e.g., zero if two hospitals are in the same region, one if in the same region group, two if into two contiguous groups, etc.…), or by the number of size quantiles separating two hospitals (e.g., zero if in the same quantile, one if in two contiguous quantiles, etc.…). A first subsample of units is generated, selecting a number of hospitals for each size-location block in order to mimic the national distribution; if the required number is not achieved for a block, hospitals from the less distant blocks that were not previously selected are also included and “assigned” to it. For a hospital, to be “assigned” means that it contributes to the total number of hospitals of a certain block even if actually it may belong to a different block, given its characteristics. The algorithm can consider either first the geographic distance and then, among ties, the hospital size distance, or vice-versa. This choice identifies two versions of the procedure: one prioritizing representativeness in geographical distribution, Distance (G), and one prioritizing hospital size, Distance (S). The QS is used in case of ties of both geographical and hospital size distances. After an initial selection, an iterative process selects random hospitals, and evaluates swaps with hospitals in the assignment status (i.e., in or out of the subsample); possible swaps are ordered by the characteristic of interest (location and size, ordered by priority) minimizing the distance between the real blocks and the assigned ones in the sample; the QS is considered for ties. The best possible swap is evaluated and accepted if it decreases the distance between the real and the assigned block for at least one of the characteristics, or improves the QS, while not impacting negatively on the fit to the target population distribution, as evaluated via log-likelihood score (see Statistical analysis).

### Statistical analysis

The QS of hospitals included in the 2016 Italian convenience sample was described using mean, median, and interquartile ranges (IQR).

The correlation between QS and hospital size was estimated using Spearman correlation index (Corr. Index) and 95% confidence intervals [95% CI] to account for non-linear monotonic relationships; the effect size of this relationship was estimated through lognormal regression and the QS Ratio [95% CI] for a 100-beds increase is reported. The relationship between QS and hospital HAI prevalence was estimated using Spearman correlation and the effect size through quasi-logbinomial regression. A quasi-likelihood model was chosen to account for the additional variance derived by the fact that patients are clustered into hospitals and therefore not independent one from another [13] as instead assumed by the binomial model, thus providing more robust results. The Risk Ratio (RR) of the HAI prevalence for a 100-point increase in the QS and QS ratio and the respective 95% CI are reported.

Both correlation and regression analyses were performed on the entire dataset and after stratification in 3 groups according to the hospital size (< 200, 200–500, ≥ 500 beds), to highlight possible non-linearities in relation to this parameter.

A simulation approach was adopted to evaluate the characteristics of the subsamples produced by the Distance, Probability, and Uniformity methods. The original convenience list was resampled by bootstrapping [14] 2000 times. For each resample ${\mathrm{S}}_{\left\{\mathrm{i}=\mathrm{1..2000}\right\}}^{\ast }$, subsamples, ${P}_{S_i^{\ast }}$, ${U}_{S_i^{\ast }}$ and ${DG}_{S_i^{\ast }}$, and ${DS}_{S_i^{\ast }}$ of size *N*_*required*_ = 55, were generated respectively by the Probability, the Uniformity, and the Distance (G and S) procedures. In addition, another subsample ${R}_{S_i^{\ast }}$ of the same size was extracted via random sampling from each resample ${S}_i^{\ast }$ of the convenience sample and used as reference; the random sample reflects the procedure used by ECDC in case countries provided data for more hospitals than requested [6].

Two indicators were used to evaluate the distributional representativeness of a sample, defined as the fit of the location-size distributions of the sample to the reference data:

- **Log-likelihood fit**: a Bayesian regularized logistic regression model with weakly informative Cauchy priors with mean zero and scale of 2.5 standard deviations [15] was trained on the national list data to predict the probability of observing a hospital from a specific location-size block given the distribution of hospitals in the country (likelihood score). The regularization allows for a smoother probability distribution along the location-size combinations, avoiding an expected probability too close to zero for underrepresented blocks. The final score is equal to the sum of the log of the likelihood score of every hospital in the sample. A lower (more negative) score indicates increasing distributional bias.
- **Spearman correlation**: The location-size block fractional frequencies were compared between the sample and the national list data using Spearman correlation. A score closer to zero indicates increasing distributional bias.

The fit of the Distance, Probability, and Uniformity subsamples was compared against the Random subsample ${R}_{S_i^{\ast }}$ through a linear regression model of the fit indicators’ value on the subsampling procedure. Since the indicator values are depending on the specification of the location-size blocks, they were computed three times after subdividing hospital size in either 5, 10, or 20 quantiles in both the subsamples and the reference data; the model is then adjusted for the number of hospital size quantiles considered as a categorical variable, and its interaction with the subsampling procedure, to factor out the possible effect of a specific subdivision criterion:

1 $$ Indicator={\beta}_{baseline}+{\beta}_{procedure} Procedure+{\beta}_{group} Group\_ size+{\beta}_{int} Procedure\ast Group\_ size+\epsilon $$

A second linear regression model with only the *β*_*baseline*_ and *β*_*procedure*_ terms was run to estimate the effect of the subsampling procedures on the following sample characteristics: the regional coefficient of variation of the number of hospitals, that is, the ratio of the standard deviation over the mean of the number of hospitals per region, reported as a percentual score; the ratio of hospitals with a number of beds lower than 200, between 200 and 500 (excluded), and equal to or higher than 500 over the total in the sample; the QS; the crude HAI prevalence, i.e. number of HAI cases over the total number of patients in the sample; the hospital average HAI prevalence, i.e., the mean of the prevalences of each hospital $\left(\frac{1}{n}\sum \limits_1^n\mathit{\Pr}\left( HAI|{Hospital}_{1..n}\right)\right)$.

For both models we reported the Effect Sizes (ES), that is the *β*_*procedure*_ coefficients describing the average difference in the outcome when using a subsampling procedure compared to a random subsample (baseline), and the Expected Value (EV) of the outcome for each procedure (i.e., *β*_*baseline*_ + *β*_*procedure*_) and for the baseline random subsample (i.e., *β*_*baseline*_). The regression model was bootstrapped 2000 times to estimate the coefficients’ sampling variability, and the median and the 90% interval of the bootstrap distribution (90% BI) are presented. We report a 90% interval instead of the canonical 95% to increase the stability of estimates [16]. Finally, the standardized effect size (stdES) was computed as the ratio of the mean over the standard deviation of the ES bootstrap resamples $\left(\frac{Mean\left(\upbeta {\ast}_{procedure}\right)}{SD\left(\upbeta {\ast}_{procedure}\right)}\right)$, to provide a dimension-less measure to compare effects among different indicators/characteristics.

All analyses and figures were produced using R v3.5.1 [17].

## Results

### Quality score

**Fig. 1:**
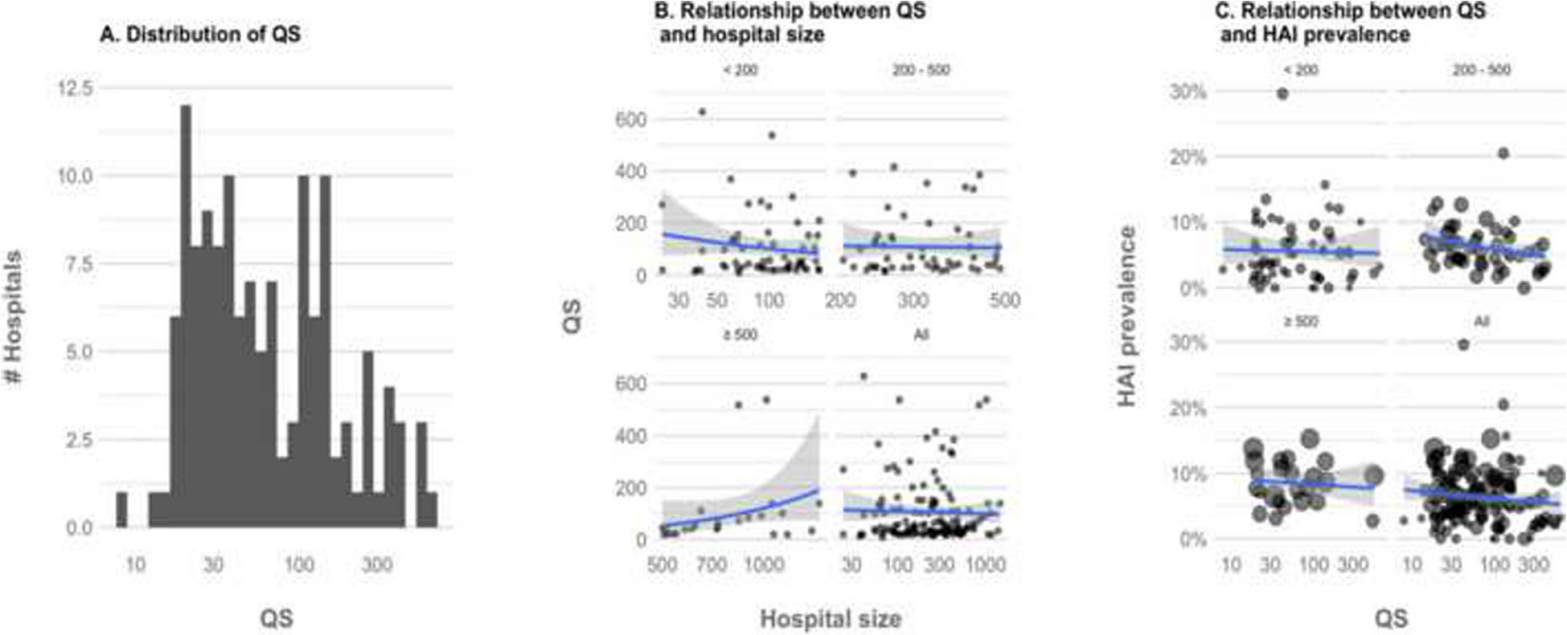
Distribution of QS and relationship with hospital size and HAI prevalence. Vertical scale in Fig. A and B transformed in Log (10). Fig. B and C show a regression line elaborated via generalized linear model with a quasi-Poisson and a quasi-binomial link function

Considering the 135 hospitals included in the convenience sample of the Italian ECDC PPS 2 study, a mean QS of 105 points was estimated, with a right-skewed distribution, as shown by a median of 52.9 points (IQR: 27.9–131); the best hospital had a score of 7.98 points and the worst of 628 points (78.7 times higher) (Fig. 1a). The study variables which mostly impacted the score were the specification of antimicrobial drug dosage (with a high number of outliers outside the central 80% of the antimicrobial specific dose distribution), the presence of warnings issued by the ECDC data acquisition software and misspecifications in the characteristics of the hospital or the ward (e.g. more single rooms then total rooms, more assessed patients than total patients), missing data regarding infection control strategies, microorganism characteristics, and the change in antimicrobial medications. The data with fewer issues were related to patient characteristics.

Our analysis did not find a definite correlation (Corr. index: 0.02, 95% CI [− 0.15; 0.19]) between QS and hospital size (Fig. 1b, Table 1) with only an appreciable positive trend for hospitals with over 500 beds (Corr. index: 0.37, 95% CI [− 0.04; 0.67]). Larger and more balanced datasets in terms of hospital size are necessary to define whether there could be a robust relationship between hospital size and QS.

**Table 1:** Relationship of QS with hospital size (number of beds) and HAI prevalence computed through Spearman correlation and regression model analysis. The correlation index [95% CI] is reported for the correlation analysis and QS Ratio and Risk Ratio [95% CI] for the regression models. Both analyses were performed on the whole dataset and after stratification by hospital size category (< 200, 200–500, ≥ 500 beds)

	Model: QS ~ Number of beds	
	Correlation index [95%CI]	QS Ratio [95%CI]
**Hospital size stratum (beds)**		
Small (< 200)	− 0.00047 [− 0.26; 0.26]	0.76 [0.41, 1.39]
Medium (200–500)	0.008 [− 0.27; 0.28]	0.95 [0.67, 1.35]
Large (≥ 500)	0.37 [− 0.038; 0.67]	1.07 [0.88, 1.3]
All	0.02 [− 0.15; 0.19]	1.02 [0.96, 1.07]
	**Model: HAI Prevalence ~ QS**	
	**Correlation index [95%CI]**	**Risk Ratio [95%CI]**
**Hospital size stratum (beds)**		
Small (< 200)	0.0025 [− 0.25; 0.26]	0.91 [0.69, 1.13]
Medium (200–500)	− 0.34 [− 0.56; − 0.071]	0.84 [0.72, 0.97]
Large (≥ 500)	− 0.067 [− 0.46; 0.35]	0.98 [0.86, 1.09]
All	−0.12 [− 0.28; 0.054]	0.93 [0.86, 1.01]

As shown in Table 1, a weak negative correlation between QS and HAI prevalence was found (Corr. index: -0.12, 95% CI [− 0.28; 0.054], HAI RR per 100 points: 0.93, 95% CI [0.86, 1.01]), but the effect is non-linear along an increase in hospital size and is present mostly in the 200–500 hospital size category (Corr. index: -0.34, 95% CI [− 0.56; − 0.071], HAI RR per 100 points: 0.84, 95% CI [0.72, 0.97]).

### Comparison of sampling procedures

As shown in Table 2, both the Distance and the Probability procedures reduced the distributional bias of the subsamples that they generated. Specifically, the Probability procedure increased the log-likelihood score by + 29 [18, 19] points (std. diff.: 4.39 SD) and the correlation coefficient by + 0.21 [0.12, 0.3] points (3.85 SD), with 100% of the bootstrapped subsamples achieving better scores than those achieved by random subsampling of the bootstrapped convenience sample. Regarding the Distance procedure, the first method, based on hospital size (S), showed the greatest improvement, with an average of + 12 points (std. diff.: 1.73 SD) of likelihood and + 0.14 points (2.51 SD) of correlation coefficient, compared to + 7.3 (1.04 SD) and + 0.095 (1.74 SD) respectively for the second method, based on location (G). The Uniformity procedure generated subsamples with a distributional bias similar or slightly worse than the subsamples generated via random sampling.

**Table 2:** Distributional fit to the reference data of the subsamples produced by the subsampling procedures and by simple random sampling, applied to the 2000 times resampled bootstrap convenience sample. The Expected Value, the Effect Size, Standardized Effect Size, and the 90% Bootstrap Intervals [90% BI] as described in the Methods, adjusted for number of quantiles chosen to compute the fit indicators. For the three sampling procedures, the percentage of bootstrap resamples in which the fit criteria improved compared to the random subsample is shown; values above 50% indicate an overall improvement

Fit Indicator	Expected Value [90% BI]	Effect Size [90% BI]	Standardized Effect Size	% of subsamples with higher values than random sampling
**Log-likelihood**				
Random	− 260 [− 270, − 250]			
Distance (G)	−250 [−260, − 240]	+ 7.3 [− 3.9, 20]	1.04	85.6%
Distance (S)	−250 [− 250, − 240]	+ 12 [1.4, 24]	1.73	91.5%
Probability	− 230 [− 240, − 220]	+ 29 [19, 40]	4.39	100%
Uniform	−260 [− 280, − 250]	−5.9 [− 18, 4.3]	− 0.88	35.9%
**Spearman Rho**				
Random	0.17 [0.076, 0.26]			
Distance (G)	0.26 [0.19, 0.34]	+ 0.095 [0.007, 0.19]	1.74	97%
Distance (S)	0.31 [0.24, 0.38]	+ 0.14 [0.051, 0.24]	2.51	98.9%
Probability	0.38 [0.33, 0.43]	+ 0.21 [0.12, 0.3]	3.85	100%
Uniform	0.16 [0.078, 0.24]	−0.013 [−0.096, 0.072]	− 0.26	51.4%

**Fig. 2:**
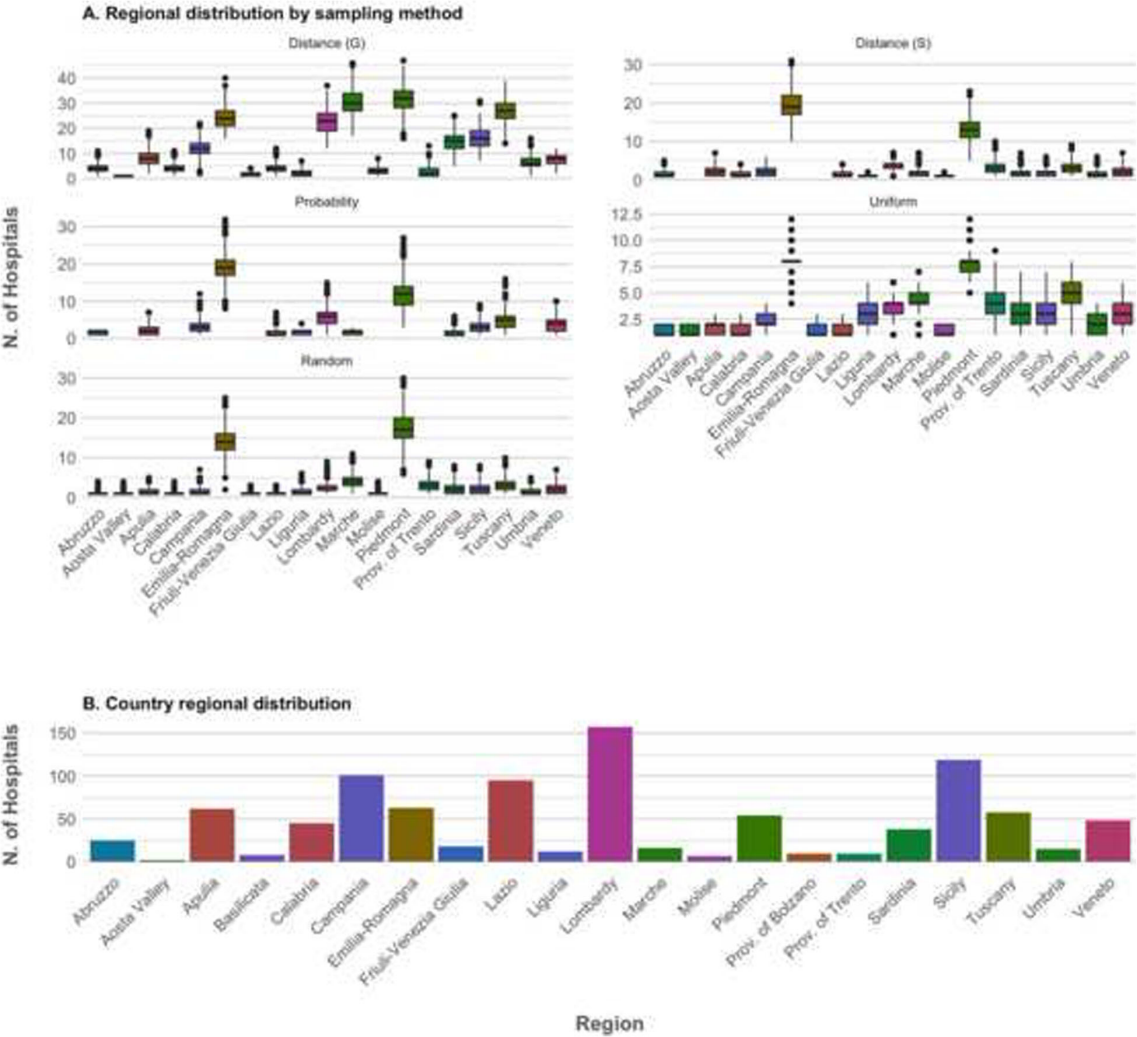
Regional distribution of hospitals after resampling and Italian regional distribution of hospitals. In **a**, boxplots show the distribution of the number of hospitals per region, after applying the considered sampling procedures (Distance, Probability, and Uniform procedures) to the bootstrapped convenience sample. The expected distribution if simple random sampling was applied is shown as reference. Panel **b** shows the actual number of hospitals per Region in Italy. Regions are color-coded for easier comparison between plots

Figure 2A depicts the regional distribution of hospitals in the subsamples produced by the considered procedures. As a reference, the Italian regional distribution of hospitals is also depicted (Fig. 2b). All procedures, apart from the Distance procedure (S), decreased the regional variability in the number of hospitals (Regional variation coefficient shown in Table 3), compensating the overrepresentation of hospitals originating from just two regions (i.e., Piedmont and Emilia Romagna) in the convenience sample (Fig. 2a). As expected, the coefficient of variation became particularly low after the Uniformity procedure (67.2% [56.1, 81.4%]), compared to 126% [98.2, 153%] in the random subsamples. Both the Distance (S) and the Probability method caused some smaller regions not to be represented in some subsamples (Fig. 2); the Probability procedure was particularly selective in excluding the most underrepresented regions.

**Table 3:** Characteristics of the subsamples. Characteristics of the subsamples produced by the subsampling procedures and by simple random sampling applied on the 2000 times resampled bootstrap convenience sample. The Expected Value, Effect Size, Standardized Effect Size and the Bootstrap Intervals [90% BI] are computed as described in the Methods. For the three sampling procedures, the percentage of bootstrap resamples in which the characteristic had a higher value than in the random subsample is shown; values above 50% indicate an overall increase while values below 50% indicate a decrease

Sample Characteristic	Expected Value 22[90% BI]	Effect Size [90% BI]	Standardized Effect Size	% of subsamples with higher values than random sampling
Regional variation coeff. (SD*100/Mean)				
Reference data	92%			
Random	125% [98.5, 152%]			
Distance (G)	73.2% [55.1, 93.4%]	−53.7% [−83, −22.5%]	−2.88	0.23%
Distance (S)	127% [101, 154%]	+ 1.94% [−31, 35.3%]	0.069	53.2%
Probability	99.1% [75.2, 127%]	−25% [−58.8, 7.12%]	−1.26	10.7%
Uniform	67.2% [56.1, 81.4%]	−58.4% [−86.1, − 31.1%]	−3.52	0%
**Small hospitals (< 200 beds, %)**				
Reference data	71.1%			
Random	43.6% [32.7, 54.5%]			
Distance (G)	43.6% [34.5, 54.5%]	+ 0% [− 12.7, 10.9%]	0.026	46.5%
Distance (S)	76.4% [72.7, 80%]	+ 32.7% [21.8, 43.6%]	4.97	100%
Probability	30.9% [21.8, 43.6%]	−12.7% [−23.6, 0%]	−1.78	3.29%
Uniform	34.5% [29.1, 41.8%]	−9.09% [−18.2, 1.82%]	− 1.32	7.19%
**Medium hospitals (≥ 200, < 500 beds, %)**				
Reference data	19.5%			
Random	38.2% [27.3, 49.1%]			
Distance (G)	36.4% [27.3, 47.3%]	−1.82% [−12.7, 10.9%]	−0.21	36.6%
Distance (S)	12.7% [9.09, 18.2%]	−25.5% [−36.4, − 14.5%]	−3.78	0%
Probability	41.8% [30.9, 52.7%]	+ 3.64% [−9.09, 14.5%]	0.47	62.3%
Uniform	38.2% [30.9, 45.5%]	+ 0% [−10.9, 10.9%]	− 0.046	41.8%
**Large hospitals (≥ 500 beds, %)**				
Reference data	9.35%			
Random	18.2% [9.09, 27.3%]			
Distance (G)	18.2% [12.7, 27.3%]	+ 1.82% [−7.27, 10.9%]	0.24	54%
Distance (S)	10.9% [10.9, 10.9%]	−7.27% [−16.4, 1.82%]	− 1.36	5.35%
Probability	27.3% [18.2, 38.2%]	+ 9.09% [0, 18.2%]	1.61	93.2%
Uniform	27.3% [20, 32.7%]	+ 9.09% [0, 16.4%]	1.74	94%
**Quality Score**				
Random	100 [79, 130]			
Distance (G)	98 [71, 130]	−6.5 [−37, 24]	−0.34	36.5%
Distance (S)	92 [68, 120]	−12 [−42, 18]	− 0.68	24.3%
Probability	68 [55, 84]	−35 [− 63, −10]	−2.21	0.9%
Uniform	99 [80, 120]	−5.1 [−34, 22]	−0.32	38.1%
**Prevalence (crude, %)**				
Random	7.44% [6.37, 8.58%]			
Distance (G)	7.23% [6.07, 8.43%]	−0.21% [−1.43, 1.02%]	− 0.3	37.3%
Distance (S)	6.98% [5.75, 8.28%]	−0.47% [−1.91, 1.01%]	−0.51	29.7%
Probability	7.87% [6.84, 8.88%]	+ 0.45% [−0.63, 1.53%]	0.69	62.7%
Uniform	7.69% [6.79, 8.45%]	+ 0.18% [−0.78, 1.29%]	0.34	75.3%
**Prevalence (hospital’s average, %)**				
Random	6.35% [5.44, 7.33%]			
Distance (G)	6.04% [5.07, 7.06%]	−0.31% [−1.34, 0.77%]	− 0.47	32.1%
Distance (S)	5.86% [4.96, 6.8%]	−0.47% [−1.49, 0.61%]	−0.72	23.8%
Probability	7.11% [6.26, 8.01%]	+ 0.8% [−0.33, 1.81%]	1.23	88.5%
Uniform	6.43% [5.61, 7.41%]	+ 0.1% [−0.9, 1.09%]	0.16	56.5%

The distribution of Italian hospitals according to size is characterized by a vast majority (71.1%) being of small hospitals (below 200 beds), 19.5% medium-sized hospitals (200 to 500 beds), and 9.35% large hospitals (more than 500 beds). The random subsamples were constituted of 43.6% [32.7, 54.5%], 38.2% [27.3, 49.1%] and 18.2% [9.09, 27.3%] of small, medium and large hospitals respectively (Table 3), indicating a strong bias towards larger hospitals in the convenience sample. The Distance (S) procedure impacted strongly on this bias, producing a distribution of small (EV: 76.4% [72.7, 80%], ES: + 32.7%), medium (12.7% [9.09, 18.2%], − 25.5%), and large (10.9% [10.9, 10.9%], − 7.27%) hospitals very similar to the Italian distribution. The distribution of large hospitals was centered around a value of 10.9%, with less than 3% of the subsamples with a lower percentage and zero subsamples with a higher percentage. The Distance (G) and Probability procedures did not provide much adjustment, compensating more for geographical bias than for hospital size. Conversely, the Uniformity procedure increased the bias, as expected, in order to achieve a more equal distribution in terms of hospital size.

All sampling procedures selected on average hospitals with higher data quality (lower QS). The QS was sensibly lower in the subsample generated using the Probability procedure, which is expected since its purpose is to reweight the probability of a hospital to be selected. Despite having a stronger influence compared to the other methods, the impact of the QS was still 7.7 times inferior to the impact of size/location in determining the final inclusion probability in the sample (analysis performed by comparing the standardized effect sizes obtained by regressing the inclusion of each hospital in the sample against either the probability of inclusion given location-size or the QS through two univariate multilevel logistic regressions).

Notably, none of the procedures significantly impacted the estimated HAI prevalence (Table 3). The largest decrease was observed after the application of the Distance (S) procedure (~ 0.5% decrease in both crude and average prevalence), as was expected considering the increment in the quota of smaller hospitals. Nevertheless, the variability of the effect was high (e.g.: 90% BI: [− 1.91, 1.01%], stdES: 0.51 SD, for the crude prevalence) and the decrease was observed only in 70% of the bootstrapped subsamples; similar results were observed for the average prevalence. On the other hand, the Distance (G) procedure produced a smaller difference in HAI prevalence whereas the Probability procedure was instead associated with a relevant increase in HAI prevalence compared with the random draw from the convenience sample.

Notably, the bootstrapped 90% intervals of both the baseline and the average changes in the prevalence estimates, in all procedures, showed a range of possible values broader than two percentage points, indicating that with such small sample sizes, wide differences between prevalence studies can be justified simply by random variation due to sampling.

## Discussion

Using the 2016 Italian PPS on HAIs as a case study , we developed a set of subsampling methodologies to improve the representativeness of epidemiologic studies when the selection of participating units is made using non-probabilistic strategies, such as convenience sampling.

Convenience sampling [8] is one of the most common non-probabilistic sampling methodologies [18, 20]. It implies that the statistical units in the sample are selected based on their availability to the researcher, in terms of physical reachability and/or willingness to be included. It is a strategy often used when it is not feasible, economically or logistically, to perform random sampling.

The major drawback of this methodology is that the selected units may not be representative of the target population, introducing distortions in the distribution of sample characteristics, and in the worst case scenario, also in the outcomes of interest [21, 22], therefore biasing estimates and decreasing the generalizability of the studies [8].

For country-level, clustered prevalence studies, convenience sampling may be simpler and more cost-effective compared to more formal sampling strategies, such as systematic sampling (as suggested by the ECDC PPS protocol), since it does not assume the compliance to participation for all (or most) of a country’s hospitals. Employing systematic sampling, for example, may be difficult when a central selection of participating hospitals is not possible, due to the specific organization of the healthcare systems (decentralized healthcare systems) or to non-willingness to enforce compliance. In Italy, regional authorities are virtually exclusively responsible for healthcare organization and delivery, within a framework provided by the central Government.

It is customary in these cases to select participating units for example among hospitals which are part of some established surveillance network [23], or to let them be chosen by regional health surveillance authorities [24].

As previously mentioned, the Italian PPS sample was generated by convenience sampling driven by regional health authorities, and this generated issues in terms of geographical and risk factor distribution. Therefore, this sample represented a perfect case for testing our methodologies, which try to measure the impact of these issues and reduce them by algorithmic subsampling.

First, we developed a score to quantify the quality of the collected data (QS) in terms of missing data and errors. In statistical practice, there is a significant corpus of literature on the negative impact of missing data on inference [25–29], especially when data is missing not-at-random [30]. The extent of the bias caused by missing data can span from a simple increase in error and uncertainty estimates, to effect magnitude and/or direction biases in risk factors analyses [30]. A common way to mitigate these issues is to employ imputation techniques [25]: these methods work either by “guessing” the missing information based on available data on the same variable (distributional imputation, e.g., taking the mean, median or mode value), or at observation level in other variables (prediction-based methods, e.g.: model-based or non-parametric imputation models), or by a combination of both. These techniques, though, are often technically and computationally demanding and may artificially reduce uncertainty or introduce bias themselves [31].

The issue of errors in the collected variables is equally relevant: they can introduce distortions in the resulting estimates and statistical associations, which are very hard to identify and account for at the analysis stage [32–34].

The score we propose considers both the amount of missing information and possible errors in data collection, weighted by the statistical relationship of the considered variables with the primary outcome of the study, i.e. the risk of HAI. This score can be used to rank and select statistical units based on the reliability (information value) of their data, thereby reducing the reliance on analytic solutions like imputation and favoring the use of the original data. The consequence is a more transparent analytic pipeline.

While applying the score to the 2016 Italian PPS data, we observed a large gradient of values, the hospitals with worse data scoring 78.7 times higher (indicating worse quality) than the one with the best (lowest) score. The analysis of the components of the score can help identify which variables have more issues: in our case, the information at the patient level was more complete than ward and hospital-level characteristics. These findings could be useful for the conduction of future surveys, by highlighting problematic variables that should be addressed when training local operators regarding data acquisition, and by revealing issues in the definitions of variables that could make their collection problematic.

When we compared the QS with HAI prevalence, we observed a slight negative association, that is, worse data quality predicted lower HAI prevalence. The relationship, albeit weak, was conserved after stratifying hospitals by their number of beds, a known predictor of HAI risk in hospitals [35]. These results could hint to an association between accuracy in data collection and quality of the HAI case finding process. It is hard to derive definitive conclusions in this regard, without having the real hospital-level prevalence and, given the small sample size, a spurious correlation cannot be ruled out. A solution could be to compute the QS of the whole “European ECDC PPS validation sample”, a group of patients in which the presence of HAI was verified by experts [6]. If the QS proves an accurate predictor of the rate of identification error without influencing the estimates, it could be then used as a tool for selecting hospitals with less biased estimates.

The second problem we tried to solve was the possible bias in the representativeness of hospitals chosen by convenience sampling (or other non-probabilistic sampling strategies). A common approach to the problem of under/overrepresentation is based on the reweighting of observational units: for example weighting observations according to the inverse of the probability of being selected to reduce disparities between subgroups [36] or based on some reference data to increase generalizability [37]. Instead, to our knowledge not much research has been devoted to a subsampling approach to the representativity issues, with a notable example in the work of Pérez Salamero Gonzálezet al [38]. We argue that a subsampling based approach may have practical advantages over a weighting based one, for example not having to manage the weights in the entirety of the analytical pipeline, or giving the possibility to clearly evaluate the impact of individual units in the final estimates. Furthermore, the common inverse-weighting based methods use only the in-sample data to estimate the weights, without relying to external reference data; this would promote more uniformity in the final sub-group representation but do not ensure generalizability of the results.

Our approach is based on two subsampling techniques (Probabilistic and Distance procedures), which, informed by country-level information, subsample hospitals generating a distribution more similar to what would have been achieved by random sampling, given a set of stratification variables. As reported in the methods, we selected geographical location and hospital size as characteristics of interest, both because these are known risk factors for HAI and increased AMU [1, 12] and since their distribution in the PPS sample was highly distorted compared to the target population. Furthermore, information about hospital location and size is easily available at the country level, compared to other important predictors (such as hospital case-mix). Nevertheless, these methods are general and can be adapted to any combination of variables for which reference data is available.

The Probability procedure uses country data to build a probability distribution given one or more variables and chooses hospitals according to it. The Distance method defines strata using the same distribution and then fills them with the hospitals in order of similarity to the strata according to a hierarchy of hospital variables: if units with the right characteristics are not available, the model selects hospitals which are as similar as possible. Finally, it further refines the sample by randomly switching hospitals in and out, updating the sample only if the switch improves the distributional fit. It can be associated to a greedy gradient search followed by a random search phase to escape possible local maxima.

We further proposed a Uniformity sampling method providing a balanced sub-sample for the considered characteristics, which may be useful for risk factor analysis and prediction models [19].

The QS is considered in all methodologies, selecting the best hospital among equivalent proposals in term of distributional fit.

Based on our dataset, both the Probability and the Distance methods sensibly decreased the distributional bias of the generated subsamples, compared to the convenience sample. This improvement highlights the possible drawbacks of non-probabilistic sampling methods and supports the necessity of adjustment before analysis. Nevertheless, it should be noted that the specific subsampling algorithm only had a relatively small effect on the final estimates of HAI prevalence, which ranged from 6.98% for the Distance method prioritizing hospital size (S), to 7.87% for the Probability method, compared to 7.44% for the convenience sample estimate. As reference, the HAI prevalence at the European level, as reported by the ECDC PPS study, was 5.9% [1]. The Distance (S) method provided the estimate clostest to the European result, perhaps because it is driven by a highly predictive risk factor (hospital size), but it is impossible, just from these results, to affirm that the Distance procedure (S) is capable of providing the more realistic estimate. An indirect way to test our methods could be using ECDC PPS data from participating countries that recruited all or a large random sample of hospitals. From these hospitals, a simulated, biased, convenience sample could be drawn, and then the three procedures could be tested to prove which sub-sampling method would be better in retrieving the original prevalence.

The variability of the bootstrapped estimates was large: all methods showed a 90% BI of more than two percentage points. This difference is quite significant in terms of burden of disease: it is greater than the variation in HAI prevalence observed among most of the countries in the European PPS study or in the same country but in surveys conducted in different years [1, 12, 35]. If we consider the estimates’ uncertainty between sampling methods, the variability is even larger, with the range of possible prevalence estimates going from 5.75% (lower bootstrap interval for the Distance (S) method) to 8.88% (upper bootstrap interval for the Probability method). These results may indicate that the differences in sampling strategies among countries and studies could explain a large portion of the observed variability in HAI prevalence. Therefore, the crude risk estimates extracted by prevalence studies should be built on larger samples and enriched by more sensitive analysis, like individual risk analysis based on patient characteristics [39, 40] or multilevel models [41–43] to factor out the hospitals’ specific contributions to risk.

Both the Probability and the Distance methods have limitations, and many improvements could be proposed.

The Probability method is the most beneficial in decreasing the global distributional bias, since it considers many variables at once, but may increase bias in specific characteristics. This happens when the amount of distortion in one characteristic is much higher than in another. The model accepts a trade-off (more distortion) on the less biased variable in order to optimize the global fit. In our case, the algorithm slightly increased the bias regarding hospital size to compensate for the highly distorted geographical distribution (i.e., two regions providing more than 50% of the total number of hospitals). This phenomenon may influence the outcome of the study itself if the bias is increased for strongly predictive variables. We indeed observed a higher HAI prevalence in the Probability sample, due to the greater quota of larger hospitals which were included by the algorithm to improve the regional representativeness (many regions provided a relative excess of large hospitals). A possible solution could be to reweight the contribution of the variables to hospital selection according to their statistical relevance with the outcome. The QS has a strong impact on the Probability method, but we showed that hospital selection is still largely driven by the country distribution of hospitals; alternatively, different weights could be attributed to the QS by exponentiating it after the rescaling (Supplementary Material S2).

The Distance method, on the other hand, allows specifying a hierarchical order for variables to be used for adjustment, so that the researcher may give priority to those more related to the outcome of interest. The drawback of this method is that it aggressively optimizes the first variable of the hierarchy, switching to the second only in case of ties; the same is the case for the third variable and so on. Therefore, its flexibility is spent mostly on few variables (primarily the first), especially if these have many possible values. This bias is compensated by a random search for solutions that improves the global fit (e.g., samples improving the fit for hospital size at the cost of a greater bias in geographical distribution are discarded), but the candidate hospitals are still evaluated using the same hierarchy.

We provide the R code for both methods (Supplementary Material S2) and we encourage researchers to experiment, improve, and adapt it for their purposes.

A general limitation of subsampling for bias correction is that it strongly impacts the sample size and, therefore, the power of studies. Only if the initial convenience sample is large, enough margin exists to compensate for the bias by subsampling. An alternative could be first to oversample the original data, increasing the size artificially, and then to produce a subsample of the original size but sub/over-sampled in order to reduce distortions.

Further validation of the sub-sampling methodologies may be pursued by taking advantage of countries with sample coverage near 100% in the European PPS sample: various distortive convenience sampling strategies may be simulated and to test how efficient are the subsampling methods in retrieving the real population prevalence.

The presented methods have been demonstrated in the context of Healthcare Associated Infection prevalence estimation in the presence of a convenience sample of acute care hospitals, but their applicability is indeed generalizable to a number of contexts and problem, wherever bias in the sample representativity and data quality issue are possibly present. For example, sample representativity isssues are common in designs that requires the opt-in of the participant units, like survey-based [44], cohort [45] and surveillance [46] studies; the same is true for errors in data collection, especially with participant self-reported data [47–49].

## Conclusions

Our analysis suggests that non-probabilistic sampling strategies might produce significant distortions in the distribution of the characteristics of a sample. This may introduce biases in the estimates which are challenging to evaluate and adjust for at the analysis stage. Therefore, we created a set of algorithms aimed at reducing this bias by producing a subsample theoretically more similar to the target population. Furthermore, we developed a quantitative score to evaluate the data quality of observational units participating in a study; this score may help in pre-analysis error individuation and correction and to improve research protocols but can also utilized to remove low quality data points if no relevant influence on the population parameters of impact is observed.

## Supplementary Information

**Additional file 1.** Rules for computing the quality score in the Italian PPS on HAI and AMU.

**Additional file 2.** Code implementation of the subsampling algorithms.

## Data Availability

Relevant code, supplementary material and simulated data is available at https://github.com/AD-Papers-Material/SubsamplingMethods. Original data is available through request at angelo.dambrosio@unito.it, conditional to approval from the involved hospitals or through request to the European Centre for Disease Prevention and Control.

## Abbreviations

AMR: Antimicrobial resistance
AMU: Antimicrobial usage
BI: Bootstrap intervals
CCM: Centro Nazionale per la Prevenzione e il Controllo delle Malattie (Natinoal Center for Disease Prevention and Control)
CI: Confidence intervals
ECDC: European Centre for Disease Prevention and Control
HAI: Healthcare-Associated Infections
IQR: Interquartile range
LRT: Likelihood ratio test
PPS: Point Prevalence survey
QS: Quality score
RR: Relative risk
SD: Standard deviation
