# Supplementary material for: Data quality assessment and subsampling strategies to correct distributional bias in prevalence studies
Source: BMC Med Res Methodol. 2021 Apr 30;21:90. doi: 10.1186/s12874-021-01277-y (PMC8088017; doi:10.1186/s12874-021-01277-y)
Supplement: Supplementary file 1 — Additional file 1. Rules for computing the quality score in the Italian PPS on HAI and AMU. [file 12874_2021_1277_MOESM1_ESM.pdf]

Supplemental Material S1. Rules for computing the quality score in the Italian PPS on HAI and AMU.

Acronyms: Healthcare Associated Infections (HAI), antimicrobial (AM), microorganism (MO), alcoholic hand rub (AHR), point prevalence study (PPS), likelihood ratio test (LRT).

General methodology

The quality score is computed through three steps:

- Definition of an importance score for each variable collected in the study.
- Enumeration of "problems" in the data for each statistical unit of reference.
- Scoring of the unit using the sum of the "problems" weighted by the importance of the involved variable.

The specific implementation of the score, the definition of the "problems" and the choosing of the statistical unit of reference are entirely aleatory, given the general framework we presented.

The score should be based on a form of statistical correlation of each variable and the primary outcome of interest. In the presence of multiple outcomes, alternative formulations may consider a mean score, a score based on a multivariate model with multiple outcomes, or a weighted score if the outcomes have different importance, with ad-hoc weights. If a statistical correlation between the outcomes and the variable is not computable, which may happen if a certain variable is consequent to the outcome itself (e.g., completeness of microbiology data in patients with HAI or antibiotic family in patients with AM prescription), an ad-hoc score may be assigned based on the importance of the variable in the study.

In our implementation, the score was computed as $10^{(1-p.value_{LRT})}$ with $p.value_{LRT}$ being the p-value of a likelihood ratio test of a univariate logistic regression model for predicting HAI risk given the variable and an intercept-only model. The resulting value is comprised between 1 and 10. The ad-hoc scores were chosen based on the quantiles of all the scores.

The enumeration of the "data problems" is a very study-specific task. The nature and precise definition of a "problem" cannot follow general rules but, in our experience, we can categorize them into:

- **Missing data**: data points that are either missing or have a placeholder implying that the data collector was not able to retrieve the information. Do not consider missing values due to non-applicability given other characteristics of the unit (e.g., missing weight at birth in non-newborns or drug type in patients not under AM therapy).
- **Inconsistencies**: unexpected values in variables connected to other variables, e.g., numerators variables with higher values than the denominator; sum of variables which is lower or higher than the total (which is collected); presence of values where non-applicable (see examples related to correct missingness).
- **Outliers**: numerical values which are unexpected given domain knowledge. The best approach should be a case by case manual evaluation. However, the identification of numerical outliers has shown in our case to be a useful heuristic: the large majority of these outliers were obvious typos or unexplained impossible values, e.g., extremely high drug dosages.
- **Data duplication**: erroneous duplication of statistical units or unit clusters, e.g. duplicated patients, duplicated wards with or without patients, etc....

Specific data problems encountered in the study

We summarize here the comprehensive list of rules applied to produce the Quality Score in this study. The statistical unit of reference for the summing up of the scores is the hospital. All the extracted values are multiplied by the importance score of the involved variables (if more than one variable, the maximum score is taken), unless differently specified. The PPS data is organized in a relational database with individual tables connected by unique IDs; the enumeration of the problems is performed at each table level and then aggregated at the hospital level.

- At the hospital level:
  - The number of missing variables.
  - The proportional difference between patients with data reported at the hospital level and total actual patients with data.
  - The proportional excess of beds included in the study to total beds in the hospital if the first value is higher than the second.
  - The proportional excess of reported occupied beds to beds assessed in the study if the first value is higher than the second.
  - The proportional excess of single-bed rooms to total rooms if the first value is higher than the second.
  - The proportional excess of single-bed rooms with toilet to total single-bed rooms if the first value is higher than the second.
  - The proportional excess of single-bed rooms with toilet to total rooms if the first value is higher than the second.
  - The proportional excess of beds with alcoholic hand rub (AHR) dispenser to assessed beds, beds included in the study or total beds in the hospital if the first value is higher than the seconds.
- At the hospital ward level:
  - The proportion of wards with missing values for each variable.
  - The number of duplicated wards (score of 1 for each duplication)
  - For the same room and AHR indicators as at the hospital level, take the mean of the relative excesses among all wards where there is data inconsistency.
  - The mean of the relative excess of healthcare workers with AHR to the total among wards if the first value is higher than the second.
- At the patient level:
  - The proportion of patients with missing values for each variable. For the proportion of missing values in the patient admission date, the importance score is the maximum (10).
- At the AM prescription level
  - The proportion of AM prescriptions with missing values for each variable.
  - Percentage of number of doses or dose strength above the 90th or below the 10th percentile for an AM, identified by the ATC code. Weighted by the median importance score
- At the HAI level:
  - The proportion of HAI with missing values for each variable. The importance score applied is 10, the maximum.

- The proportion of HAI for which the involved microorganism (MO) is not reported even if the microbiological identification was necessary in the case definition the HAI (i.e., Pneumonia with positive microbiology, urinary tract infection with positive microbiology, laboratory-confirmed bloodstream infection not central venous catheter-associated, central venous catheter-associated infection with positive microbiology, peripheral venous catheter-associated infection with positive microbiology, Clostridium-associated infection). The importance score applied is the maximum value in the overall score distribution.
- The proportion of HAI with missing AM susceptibility results for the relevant MO. The importance score applied is 10, the maximum.

- Errors and warning reported by the HelicsWin ECDC software for each hospital:
  - HelicsWin reports an error if a patient is labeled as having a HAI or being under AM treatment but then no data in the respective database tables are found. The proportion of such patients is used for the score, multiplied by the maximum value in the overall score distribution.
  - HelicsWin reports a number of warnings for each patient, ward, and hospital. The proportion of unit with each warning type is recorded, weighted by the median value of the score distribution for ward and hospital-related warning and by the maximum score for patient-related warnings. Among the warnings: dates' inconsistencies, outliers for some values like patient-days and health-care workers, data inconsistencies (numerator higher than denominator), recorded wards with no associated patients, etc.
