# Supplementary material for: Data quality assessment and subsampling strategies to correct distributional bias in prevalence studies
Source: BMC Med Res Methodol. 2021 Apr 30;21:90. doi: 10.1186/s12874-021-01277-y (PMC8088017; doi:10.1186/s12874-021-01277-y)
Supplement: Supplementary file 2 — Additional file 2. Code implementation of the subsampling algorithms. [file 12874_2021_1277_MOESM2_ESM.pdf]

Supplemental Material S2. Code implementation of the subsampling algorithms.

Acronyms: Healthcare Associated Infections (HAI), Antimicrobial/s (AM), Antimicrobial Usage (AMU), Microorganism (MO), Alcoholic Hand Rub (AHR), Point Prevalence Study (PPS), Likelihood Ratio Test (LRT).

Introduction

We implemented three algorithms, defined "Distance procedure", "Probability procedure", and "Uniformity procedure", in order to produce subsamples with specific distributional properties starting from an initial data collection which is possibly biased in terms of generalizability to the target population. In the following document we present the R code to implement the algorithms. The specific implementation described here is focused on the Italian PPS study on HAI/AMU prevalence in acute care hospitals but can be easily translated to different settings.

The methods try to change the distributional characteristics of a sample by producing a subsample whose units are chosen using information on some characteristics of interest. Two of these methods, the Distance procedure and the Probability procedure, aim at generating a representative sample of a target population, by using reference data at the population level with information on the distribution of relevant characteristics. On the contrary, the Uniformity procedure generates a sample which is uniform in relation to such characteristics, therefore it does not require population reference data.

This document and all the relative material is available at https://github.com/AD-Papers-Material/SubsamplingMethods.

Input data

The algorithms take as input a database with a statistical unit for each row and the characteristics of interest as columns. In addition, a column with a unique ID for each unit is needed. Our algorithms also accept a Quality Score (QS, cfr. Methods and Supplementary material S1) as additional characteristic.

The Probability and Distance procedures require population level reference data with information for on same characteristics. If individual unit data is not available at the population level, simulated data can be generated starting from the joint distribution of the considered characteristics, ensuring that the simulated dataset is large enough to limit random variation.

Our study data use acute care hospitals as observational units and hospital size (number of acute care beds) and region of location as characteristics of interest. We also used the official Italian national database of acute care hospital as population level data. We provide simulated sample data and the population level data for the testing of the procedures at https://github.com/AD-Papers-Material/SubsamplingMethods. These two datasets also provide hospital size grouped in three categories as used in the manuscript.

```
# Simulated sample data retaining the real sample characteristics
str(Sample.Data, vec.len = 3)
#> 'data.frame':    143 obs. of  5 variables:
#>  $ Region: chr  "regione piemonte" "regione piemonte" "regione piemonte" ...
#>  $ Beds  : int  183 172 157 179 85 133 189 182 ...
#>  $ QS    : num  101 628 147 109 ...
#>  $ Class : chr  "< 200" "< 200" "< 200" ...
#>  $ Code  : int  1 2 3 4 5 6 7 8 ...

# Official list of Italian acute hospitals, updated to 2016
```

```
str(Reference.Data, vec.len = 3)
#> 'data.frame':    963 obs. of  4 variables:
#>  $ Code  : int  10007 10010 10012 10612 10653 10655 10003 10011 ...
#>  $ Beds  : int  258 73 22 105 9 96 337 368 ...
#>  $ Class : chr  "200 - 500" "< 200" "< 200" ...
#>  $ Region: chr  "regione piemonte" "regione piemonte" "regione piemonte" ...
```

General aspects and notation

The procedures at the moment can utilize only **two characteristics** at once and these need to be **categorical variables**. Therefore continuous characteristics (specifically, hospital size in this case) are discretized in quantiles by the algorithms before use. The number of quantiles to split continuous features into is an input to the algorithms.
The hospitals are then grouped into *blocks* according to the characteristics of interest: in this study, we used *location/hospital size blocks*, defined by the Italian region where hospitals are ($Region$) and the quantile of the number of their acute beds ($HSize$). The region and the hospital size quantile allow the definition of a joint discrete probability distribution which is used by the algorithms.
For each block $block_i$ a probability $p_i = Pr(hospital|Region, HSize)$ is defined, both for the sample ($p_{i,sample}$) and the target population ($p_{i,country}$) which indicates the fraction of hospitals in a block over the total. All the algorithms are constrained by a parameter $N_{required}$ which defines the size of the final subsample.
The algorithms employ the Quality Score QS as a further discriminant in sampling; to avoid using it without code modification it is sufficient to assign the same value (a positive number) to all units. Note that a lower QS implies better data quality.

Uniformity procedure

This procedure subsamples hospitals so as to obtain an equal number of units in every block, by iteratively choosing one hospital from each block. This is the general implementation:

- a candidate list is created from the original sample;
- the hospitals in the list are permuted randomly or ordered in ascending order by QS;
- the first hospital is selected;
- all other hospitals belonging to the same block as the selected hospital are removed from the candidate list;
- the process is repeated until there are still available blocks in the candidate list;
- once one hospital from each blocks has been chosen, the hospitals from all the blocks are made available again in the list, apart from those already selected in the sample;
- continue until $N_{required}$ is reached.

For the uniform procedure, we discretized the number of beds into 4 quantiles only, since it was less relevant to build a precise discrete probability distribution.

```
uniform.sampling <- function(Input.Sample, n.required, n.quantiles = 4, use.QS = T){

    library(dplyr)
    library(Hmisc)
```

```r
    # Prepare data by discretizing continuous variables like the number of acute
    # beds and by changing QS to a fixed value if not to be used
    Hospitals <- Input.Sample %>%
        transmute(
            Code, Region,
            Beds = Hmisc::cut2(Beds, g = n.quantiles),
            QS = if (use.QS) QS else 1)

    Selected.hospitals <- c()
    Candidates <- data.frame()

    for (i in 1:n.required) { # Until n.required is reached..

        # If the candidate list is empty, rebuilt it from the non-selected hospitals
        if (nrow(Candidates) == 0) {
            Candidates <- Hospitals %>%
                # Remove already selected hospitals
                filter(!(Code %in% Selected.hospitals)) %>%
                # Permute order, useful only if QS is not used
                sample_frac() %>%
                # Arrange by QS ascending, best hospitals first
                arrange(QS)
        }

        # Extract the first hospital of the temporary list and add it to the list of
        # selected hospitals
        Extracted.hospital <- Candidates[1,]
        Selected.hospitals <- c(Selected.hospitals, Extracted.hospital$Code)

        # Remove from the temporary list all hospitals in the same location/size block
        # of the extracted hospital
        Candidates <- Candidates %>%
            filter(
                !(Region %in% Extracted.hospital$Region),
                !(Beds %in% Extracted.hospital$Beds)
            )
    }

    # Filter the initial data by the selected hospital codes
    Input.Sample %>% filter(Code %in% Selected.hospitals)
}

## Examples

# Create a subsample of 55 hospitals using the QS
# subsample.uniform(Sample.Data, n.required = 55)

# The same but this time hospitals are chosen randomly
# subsample.uniform(Sample.Data, n.required = 55)
```

Probability procedure

This algorithm uses information from a population level list (Reference Data) to built a discrete probability distribution of the target population, which is then used to drive the creation of a representative subsample. Hospitals are selected proportionally to the relative weight of the block to which they belong, in term of number of hospitals over the total. The QS is used to weight such representativeness. The weight of the QS itself can be tuned.

- the blocks are identified in the Reference Data and for each block i the probability $p_{i,country} = Pr(hospital|Region, Hospital.Size) = \frac{N_{hospitals}|block_i}{N_{hospitals}}$ is computed as the proportion of hospitals in the block over the total;
- these probabilities are assigned to the relative blocks in the sample;
- a score is computed for each hospital j as $score_j = p_{j,country}(1 - scaled.QS_j)^w$ where:
  - $p_{j,country}$ is the probability of the block of the hospital j at the country level;
  - $scaled.QS_j$ is the QS of the hospital j after that all hospital QS have been rescaled to the range [0,1], with 1 representing the worst quality score and 0 the best. The term $(1 - scaled.QS_j)$ reweights the sampling probability of a hospital using the quality of its data;
  - w allows scaling the importance of the QS in the selection, with $w = 0$ removing its influence;
- finally, $score_j$ is used to arrange the hospitals and the first $N_{required}$ with the higher score get selected. In alternative, the score can be used as a weight for selecting the hospitals by weighted random sampling.

```
probability.sampling <- function(Input.Sample, Reference.Data, n.required,
    n.quantiles = 10, QS.weight = 1,
    method = c('arrange', 'random')){

    library(dplyr)
    library(Hmisc)
    library(magrittr)
    library(scales)

    method <- match.arg(method)

    # Definition of quantiles in the distribution of number of beds according to
    # reference data
    quantiles <- quantile(Reference.Data$Beds, seq(0, 1, length.out = n.quantiles)) %>%
        round()

    # Definition of the distributional blocks at the target population level
    P_country <- Reference.Data %>%
        count(Block = Hmisc::cut2(Beds, quantiles) %>% paste('-', Region)) %>%
        mutate(Prob = n / sum(n)) %>%
        with(magrittr::set_names(Prob, Block))

    Selection <- Input.Sample %>%
        mutate(
            # Identification of the country level blocks in the sample
            Block = Hmisc::cut2(Beds, quantiles) %>% paste('-', Region),
            # Association of P_country to the hospital in the sample
```

```r
            Prob = P_country[Block],
            # Creation of the quality weight after rescaling of the QS
            QS.rescale = 1 - scales::rescale(QS),
            # Definition of the final score
            Score = Prob * QS.rescale^QS.weight
        ) %>%
        filter(!is.na(Score)) # Remove blocks that do not appear in the national list

    if (method == 'arrange') {
        Selection %>%
            arrange(desc(Score)) %>%
            head(n.required)
    } else {
        slice_sample(Selection, n = n.required, weight_by = Score)
    }
}

## Examples

# Create a subsample of 55 hospitals
# subsample.probability(Sample.Data, Reference.Data, n.required = 55)

# Set QS.weight to 0 to not use the QS in the sampling
# subsample.uniform(Sample.Data, Reference.Data, n.required = 55, QS.weight = 0)

# Create a subsample of 55 hospitals with weighted random selection
# subsample.probability(Sample.Data, Reference.Data, n.required = 55, method = 'random')
```

Distance procedure

As with the Probability procedure, the aim of this algorithm is to produce subsamples that are representative of the target population. The advantage of this procedure is that it is particularly appropriate when the original sample is particularly distorted in relation of the characteristics of interest. That is, some blocks are too underrepresented compared to target population and therefore the Probability procedure cannot find enough units to reproduce their representativeness at the population level.
This procedure tries to solve the problem by taking units from blocks which are similar to the underrepresented ones and have an excess of hospitals: if a block cannot provide enough units as required, samples are collected from blocks with similar characteristics. *Similarity* is defined through ad-hoc distance measures between blocks for each characteristics.
The procedure considers the sample characteristics of interest sequentially, implicitly giving more weight to one or another. Such priority can be passed as an argument.

This algorithm is more complex than the previous two procedures. Here's the general implementation:

- for each i block the expected number of sampled units give population data is computed as $N_{i,expected} = \texttt{Round}(p_{i,country} \times N_{required})$, with $p_{i,country}$ being the target population level representativeness of a location/size block, as defined above;
- in case $N_{required}$ is not reached, i.e., $(\sum_{i=1}^{n} N_{i,expected}) < N_{required}$, the $N_{i,expected}$ of specific blocks is increased by one unit until $N_{required}$ is achieved. Units are added to blocks for which the difference between $p_{i,country} \times N_{required}$ not rounded and $N_{i,expected}$ (rounded) is higher than zero $((p_{i,country} \times N_{required}) - N_{i,expected} > 0)$, starting by the highest values, closer to be rounded up;

- for each block, starting by those requiring more hospitals, $N_{i,sample}$ hospitals belonging to it are selected;

- if $N_{i,sample} < N_{i,expected}$ (i.e., the block is underrepresented), hospitals from similar blocks are *assigned* to it and included in the subsample, becoming not available for the other blocks. The final number of hospitals assigned to a block is $N_{i,assigned}$ and may comprise hospitals actually belonging to other blocks: for example a hospital from the Piedmont region could be inserted in the subsample but assigned to the Lombardy region since it is a close region. Similarity is computed via characteristic-specific algorithms (check the R code online at https://github.com/AD-Papers-Material/SubsamplingMethods), and is evaluated with a priority chosen by the user: either hospital size similarity is considered followed by location similarity or vice-versa. In case of ties, the QS is used while random selection if also the QS is equal.

At this point, an initial subsample is achieved, with $N_{required}$ hospitals each representing a block (not necessarily the actual block they belong to); hospitals not included in the sample are labeled as "unassigned" to any block. The initial subsample may have a bias due to the order in which blocks are evaluated, which depends on $N_{i,expected}$: some blocks may not access their hospitals anymore since they were already assigned to a block evaluated earlier.
A *random search* attempts to reduce this bias by finding solutions which decrease the overall distance between the *assigned* and real hospital blocks, while improving the fit to the population data.
This is done by randomly selecting a hospital, inside or outside the subsample, and evaluating possible swaps with other hospitals. All the swaps are evaluated and the best one, in term of lower real-assigned block distance, is accepted if the overall subsample distance for one of the characteristic or the overall QS decrease, and the swap does not impact negatively on its target population representativeness:

- for a chosen number of iterations, a random hospital is chosen and a set of candidate hospitals to be swapped is generated from those with the opposite assignment status (i.e.: if the hospital is included in the subsample, than the candidates are chosen among those not included and vice-versa);

- For each candidate swap, the assigned region and size class are inverted with those of the randomly selected hospital and the distance between the new assigned block and the real block of the hospitals is computed. Unassigned hospitals are always at distance of one from their real block;

- The candidate swap chosen is the one that minimizes the sum of distances arranged by size and location (in order according to priority), as long it provides an improvement in the distance in at least one of the characteristics or a decrease of the overall QS in the subsample. For example (considering only location for simplicity), if the random chosen hospital is from the Tuscany region but is assigned to Sicily (distance: 2) and the candidate is a hospital from Sicily but unassigned (distance: 1), the swap would unassign out of the subsample the first hospital (distance: 1) and include the other assigning it to Sicily (distance: 0): the final distance is (1 + 0) - (2 + 1) = -2, therefore implying an overall improvement of the distances;

- the swap is finally accepted if the target population representativeness of the subsample after the swap does not decrease. The representativeness is evaluated through a log-likelihood based score as described in the statistical analysis section of the manuscript. The code to compute such score is available online.

```
subsample.distance <- function(Input.Sample, Reference.Data, n.required,
     n.quantiles = 10, priority = c('size', 'location'),
     method = c('logLik', 'spearman'), reallocate = T,
     realloc.steps = 2000, steps.to.try = 2 * n.required){

    library(dplyr)
    library(Hmisc)
    library(pbapply)
    library(parallel)
```

```r
priority <- match.arg(priority)
method <- match.arg(method)

# Definition of quantiles in the distribution of number of beds according to
# reference data
quantiles <- quantile(Reference.Data$Beds, seq(0, 1, length.out = n.quantiles)) %>%
    round()

Hospitals <- Input.Sample %>%
    transmute(
        Code, QS,

        # Hospital size is quantized according to reference data and location/size
        # blocks are defined
        Region,
        Size.class = Hmisc::cut2(Beds, quantiles),
        Block = paste(Size.class, '-', Region),

        # To each hospital is assigned a fictitious region and size class, the
        # initial value is 'unassigned' which means not selected in the sample
        Region.assigned = 'unassigned',
        Size.class.assigned = factor('unassigned',
            levels = c(levels(Size.class), 'unassigned')),

        # The distance associated to the 'unassigned' status is 1
        Distance = 1,
        Size.diff = 1
    )

# Blocks are identified in the reference data too
Reference.Data <- Reference.Data %>% mutate(
    Size.class = Hmisc::cut2(Beds, quantiles),
    Block = paste(Size.class, '-', Region)
)

# For each block the expected number or required hospital is computed,
# translating the target population proportion to the required sample size
Blocks <- count(Reference.Data, Block, name = 'N.country') %>%
    mutate(
        F.expected = N.country/sum(N.country) * n.required,
        N.expected = round(F.expected),

        # If the required sample size is not reached, additional units are added
        # to blocks closer to be rounded up
        N.expected = if (sum(N.expected) == n.required) N.expected else {
            delta <- (F.expected - N.expected)
            rank <- row_number(-delta) # rank blocks by fractional part
            missing <- 1:(n.required - sum(N.expected))
            case_when(
                rank %in% missing & delta > 0 ~ N.expected + 1,
                T ~ N.expected
            )
        }
```

```r
    ) %>%
    # Join the expected block numerosity with the Reference data, to associate
    # information about region and size class
    left_join(Reference.Data[,c('Region', 'Size.class', 'Block')], by = 'Block') %>%
    distinct %>%
    # Blocks that need more hospital first
    arrange(desc(F.expected))

## Reservoir of available hospitals to assign
Available.hospitals <- Hospitals %>%
    slice_sample(prop = 1)

# First assignment: associate hospitals to block until N_expected is reached;
# if not enough hospitals for a block are available in the sample, uses
# hospitals from "similar" blocks.
Hospitals.reassigned <- pblapply(which(Blocks$N.expected != 0), function(i) {
    Block <- Blocks[i,]

    if (Block$N.expected == 0) return(NULL) # No hospitals required for this block

    # Compute "distances" of the block with all hospitals
    Available.hospitals$Distance <- get.location.distance(
        rep(Block$Region, nrow(Available.hospitals)),
        Available.hospitals$Region
    )
    Available.hospitals$Size.diff <- get.onedim.distance(
        rep(Block$Size.class, nrow(Available.hospitals)),
        Available.hospitals$Size.class
    )

    # Arrange hospitals by distance from the block, prioritizing the
    # chosen characteristic. Hospitals belonging to the block will have
    # zero distance and will be prioritize. QS is used in case of ties
    Selected.hospitals <- Available.hospitals %>% {
        if (priority == 'size') {
            arrange(., Size.diff, Distance, QS)
        } else {
            arrange(., Distance, Size.diff, QS)
        }
    } %>%
        # Select the N_expected hospitals for the block, ordered by distance.
        head(Block$N.expected) %>%
        # "Assign" the block size location to the selected hospitals
        mutate(
            Region.assigned = !!Block$Region,
            Size.class.assigned = !!Block$Size.class
            )

    # Remove the selected hospitals from those still available for sampling
    Available.hospitals <<- Available.hospitals %>%
        filter(!(Code %in% Selected.hospitals$Code))

    Selected.hospitals
```

```r
}) %>% bind_rows()

score <- get.distr.fit(Hospitals.reassigned, Reference.Data, method = method)
message('First distribution score: ', score)

# Rebuild the dataset with assigned and not hospitals
Hospitals <- bind_rows(
    Hospitals.reassigned,
    Hospitals %>% filter(!(Code %in% Hospitals.reassigned$Code))
)

if (reallocate) {
    message('Reallocation')

    # Windows doesn't support forking for parallelization, and socketing is
    # actually slower
    options(mc.cores =  if (Sys.info()[['sysname']] == 'Windows') {
        1
    } else parallel::detectCores())

    steps.wo.improvements <- 0

    # Initiate random swapping of hospitals to improve the fit
    pblapply(1:realloc.steps, function(step) {

        steps.wo.improvements <<- steps.wo.improvements + 1

        # iI too many steps are gone without improvement, stops
        if (steps.wo.improvements > steps.to.try) {
            return()
        }

        # Select a random hospital
        Evaluated.hospital <- slice_sample(Hospitals, n = 1)

        # Evaluate swaps with hospital in the opposite assignment status
        Candidate.swaps <- Hospitals %>%
            filter(if (Evaluated.hospital$Region.assigned == 'unassigned') {
                Region.assigned != 'unassigned'
            } else Region.assigned == 'unassigned')

        # Evaluate all candidate swaps, computing the change in distance between
        # the assigned and the actual block. mclapply allows parallel looping on
        # Unix OS, but reverts to lapply on Windows
        Best.swap <- mclapply(1:nrow(Candidate.swaps), function(i) {
            Candidates <- bind_rows(Evaluated.hospital, Candidate.swaps[i,])

            # Compute the distances after swapping
            Candidates$swapped.dist <- get.location.distance(
                Candidates$Region, rev(Candidates$Region.assigned)
            )
            Candidates$swapped.size.diff <- get.onedim.distance(
                Candidates$Size.class, rev(Candidates$Size.class.assigned)
```

```r
        )
        Candidates$swapped.QS <- rev(Candidates$QS)

        data.frame(
            Evaluated = Candidates$Code[1],
            Candidate = Candidates$Code[2],

            # Compute the delta in the distances, a negative value
            # means improvement
            delta.location = with(Candidates, sum(swapped.dist) - sum(Distance)),
            delta.size = with(Candidates, sum(swapped.size.diff) - sum(Size.diff)),
            delta.QS = with(
                filter(Candidates, Region.assigned != 'unassigned'),
                swapped.QS - QS
            )
        )
    }) %>% bind_rows() %>%
        # Arrange the swaps according to priority
        {
            if (priority == 'size') {
                arrange(., delta.size, delta.location, delta.QS)
            } else {
                arrange(., delta.location, delta.size, delta.QS)
            }
        } %>% head(1) # And select the best swap

    # If at least one of the distances real/assigned blocks is improved proceed
    if (with(Best.swap, delta.location < 0 | delta.size < 0 | delta.QS < 0)) {

        # Select the hospitals of the best swap and invert their assigned
        # blocks. Then recompute the distances
        Best.swap.hospitals <- Hospitals %>%
            filter(Code %in% unlist(Best.swap[,1:2])) %>%
            mutate(
                across(c(Region.assigned, Size.class.assigned), rev),
                Distance = get.location.distance(Region, Region.assigned),
                Size.diff = get.onedim.distance(Size.class, Size.class.assigned)
            )

        # Remove the swapped hospitals from the sample and add them again with
        # the updated assignment
        Hospital.proposal <- Hospitals %>%
            filter(Code %nin% Best.swap.hospitals$Code) %>%
            rbind(Best.swap.hospitals)

        # Compute the fit with the target population before and after the swap
        score_before <- get.distr.fit(
            filter(Hospitals, Region.assigned != 'unassigned'),
            Reference.Data, method = method)
        score_after <- get.distr.fit(
            filter(Hospital.proposal, Region.assigned != 'unassigned'),
            Reference.Data, method = method)
```

```r
                # If the the swap didn't decrease the fit, the swap is accepted
                if (score_after >= score_before) {

                    message('\nStep: ', step)
                    message('Improvement after ', steps.wo.improvements, ' steps')
                    message('Score after reassignment:', score_after)

                    steps.wo.improvements <<- 0

                    Hospitals <<- Hospital.proposal
                }
            }
        })

    }

    # Return the subsampled data
    Input.Sample %>%
        filter(Code %in% filter(Hospitals, Region.assigned != 'unassigned')$Code)
}

## Examples

# Create a subsample of 55 hospitals prioritizing hospital size, without
# reallocation after the initial sampling

# subsample.distance(Sample.Data, Reference.Data, n.required = 55,
#   reallocate = F)

# This time prioritize location and perform reallocation. (Warning: it takes
# time!)

# subsample.distance(Sample.Data, Reference.Data, n.required = 55,
#     priority = 'location')
```
